# Dietary Supplementation with Olive Oil or Fish Oil and Vascular Effects of Concentrated Ambient Particulate Matter Exposure in Human Volunteers

**DOI:** 10.1289/ehp.1408988

**Published:** 2015-05-01

**Authors:** Haiyan Tong, Ana G. Rappold, Melissa Caughey, Alan L. Hinderliter, Maryann Bassett, Tracey Montilla, Martin W. Case, Jon Berntsen, Philip A. Bromberg, Wayne E. Cascio, David Diaz-Sanchez, Robert B. Devlin, James M. Samet

**Affiliations:** 1 Environmental Public Health Division, National Health and Environmental Effects Research Laboratory, U.S. Environmental Protection Agency, Research Triangle Park, North Carolina, USA; 2 Department of Medicine, University of North Carolina at Chapel Hill, Chapel Hill, North Carolina, USA; 3 TRC Environmental Corporation, Raleigh, North Carolina, USA

## Abstract

**Background:**

Exposure to ambient particulate matter (PM) induces endothelial dysfunction, a risk factor for cardiovascular disease. Olive oil (OO) and fish oil (FO) supplements have beneficial effects on endothelial function.

**Objective:**

In this study we evaluated the potential efficacy of OO and FO in mitigating endothelial dysfunction and disruption of hemostasis caused by exposure to particulate matter (PM).

**Methods and Results:**

Forty-two participants (58 ± 1 years of age) received either 3 g/day of OO or FO, or no supplements (naive) for 4 weeks prior to undergoing 2-hr exposures to filtered air and concentrated ambient particulate matter (CAP; mean, 253 ± 16 μg/m^3^). Endothelial function was assessed by flow-mediated dilation (FMD) of the brachial artery preexposure, immediately postexposure, and 20 hr postexposure. Levels of endothelin-1 and markers of fibrinolysis and inflammation were also measured. The FMD was significantly lower after CAP exposure in the naive (–19.4%; 95% CI: –36.4, –2.3 per 100 μg/m^3^ CAP relative to baseline; *p* = 0.03) and FO groups (–13.7%; 95% CI: –24.5, –2.9; *p* = 0.01), but not in the OO group (–7.6%; 95% CI: –21.5, 6.3; *p* = 0.27). Tissue plasminogen activator levels were significantly increased immediately after (11.6%; 95% CI: 0.8, 22.2; *p* = 0.04) and 20 hr after CAP exposure in the OO group. Endothelin-1 levels were significantly increased 20 hr after CAP exposure in the naive group only (17.1%; 95% CI: 2.2, 32.0; *p* = 0.03).

**Conclusions:**

Short-term exposure to CAP induced vascular endothelial dysfunction. OO supplementation attenuated CAP-induced reduction of FMD and changes in blood markers associated with vasoconstriction and fibrinolysis, suggesting that OO supplementation may be an efficacious intervention to protect against vascular effects of exposure to PM.

**Citation:**

Tong H, Rappold AG, Caughey M, Hinderliter AL, Bassett M, Montilla T, Case MW, Berntsen J, Bromberg PA, Cascio WE, Diaz-Sanchez D, Devlin RB, Samet JM. 2015. Dietary supplementation with olive oil or fish oil and vascular effects of concentrated ambient particulate matter exposure in human volunteers. Environ Health Perspect 123:1173–1179; http://dx.doi.org/10.1289/ehp.1408988

## Introduction

Epidemiological studies have demonstrated an association between exposure to ambient particulate matter (PM) at concentrations currently found in major metropolitan areas and a broad range of adverse cardiovascular outcomes (U.S. EPA 2009). A recent scientific statement from the American Heart Association concluded that short-term elevations in PM concentrations are capable of triggering acute coronary syndrome and stroke, worsening heart failure, and provoking arrhythmias among individuals with preexisting heart disease (Brook et al. 2010).

Ambient PM is a complex and variable mixture of particles of varying size that are classified into coarse (PM_2.5–10_; from 2.5 μm to 10 μm in aerodynamic diameter), fine (PM_2.5;_ < 2.5 μm), and ultrafine (PM_0.1_; < 0.1 μm) particles. In the present study, research volunteers were exposed to PM_2.5_ particles concentrated from the air (CAP) in Chapel Hill, North Carolina. Short-term controlled human exposure to PM_2.5_ increases blood pressure (BP) and impairs endothelial function (Brook et al. 2009) in young healthy adults. Controlled exposure to diesel exhaust, a component of ambient PM, has been shown to affect fibrinolysis (Mills et al. 2005). A recent epidemiological study has associated long-term exposure to PM with decreased endothelial function (Krishnan et al. 2012). Moreover, PM_2.5_ exposure in the Chapel Hill airshed was associated with immediate endothelial dysfunction in diabetic patients (Schneider et al. 2008). In addition, several controlled human exposure studies have suggested that air pollution exposure increases BP during (Brook et al. 2009; Urch et al. 2005) and immediately after (Mills et al. 2005) PM exposure, and ambient PM_2.5_ levels have been associated with higher BP in epidemiological studies (Auchincloss et al. 2008). However, the mechanisms of these vascular effects associated with exposure to ambient PM are still not well understood.

Endothelial dysfunction is a critical early event in the development of atherosclerosis (Ross 1993). Endothelial dysfunction is defined as an imbalance between vasodilating and vasoconstricting substances produced by and acting on the endothelium. Endothelial dysfunction results in elevated expression of chemokines, cytokines, and adhesion molecules that promote smooth muscle cell growth, platelet and leukocyte adhesion, thrombosis, and vascular inflammation. Endothelial dysfunction induced by air pollution exposure has been implicated as an important mechanism in the development of cardiovascular diseases (Brook et al. 2010; Krishnan et al. 2013). Inflammation and oxidative stress mediate the adverse cardiovascular effects of air pollution exposure (Brook et al. 2010). Dietary supplements such as olive oil (OO) and fish oil (FO) have been shown to have antioxidant and anti-inflammatory effects (Moreno and Mitjavila 2003) that might offer protection against air pollution exposure. Consumption of OO, a principal component of the Mediterranean diet, has been shown to improve endothelial function and blood lipid profile (Zern and Fernandez 2005), decrease platelet aggregation (Delgado-Lista et al. 2011), and reduce vascular inflammation (Esposito et al. 2004). In particular, some components of OO, such as polyphenols (Moreno-Luna et al. 2012; Zern and Fernandez 2005) and oleic acid (Perona et al. 2006), have been shown to have beneficial effects on endothelial function. Therefore, OO supplementation is a potential candidate for use as a protective intervention against the adverse vascular effects of PM exposure. Dietary supplementation with FO has been shown to improve endothelial function in patients who smoke tobacco or who have elevated insulin, glucose, or triglyceride levels (Duda et al. 2009a; Saravanan et al. 2010). However, the effects of OO and FO on vascular responses to air pollution exposure have not been examined specifically.

In the present study, we assessed the acute effects of controlled exposure to ambient air PM_2.5_ on vascular endothelial function, blood pressure, and fibrinolysis and inflammation in healthy middle-aged human volunteers, and evaluated the efficacy of a 4-week regimen of dietary supplementation with OO or FO as a means to mitigate vascular effects induced by PM exposure.

## Methods

*Study participants*. Forty-two participants ranging in age from 50 to 72 years (mean 58 ± 1 years) were enrolled in the study. None had a history of heart disease, uncontrolled hypertension, pulmonary disease, diabetes mellitus, hypercholesterolemia, or active allergy, and none had smoked for the past year. Participants were not taking n-3 or n-9 fatty acid (FA) supplements, anti-inflammatory drugs [e.g., nonsteroidal anti-inflammatory drugs (NSAIDs)], or antioxidant supplements (e.g., beta-carotene, selenium, vitamin C, and vitamin E). All participants were instructed to avoid food sources of n-3 and n-9 FA for 6 weeks and refrain from using any NSAIDs for 2 weeks prior to each exposure. They were also asked to abstain from alcohol and caffeine and adhere to a low-fat diet for 24 hr before exposures. The Biomedical Institutional Review Board at the University of North Carolina at Chapel Hill and the U.S. Environmental Protection Agency approved the study protocol, recruitment materials, and consent forms. All study participants gave informed consent and received monetary compensation for their participation.

*Study design*. All exposures were conducted in the U.S. Environmental Protection Agency Human Studies Facility (HSF) on the medical campus of the University of North Carolina at Chapel Hill as described previously (Tong et al. 2012). At the end of 28 days of dietary supplementation, each participant was exposed first to filtered air and then returned to the HSF the next morning to undergo exposure to CAP. The participants were blinded to the exposure. The exposures were conducted at the same time of the day and on the same 2 days of the week. Participants were exposed for 2 hr through a face mask in an exposure chamber in which temperature and humidity were controlled. They remained at rest in a seated position throughout the exposure.

The following procedures were performed on each participant beginning at approximately 0800 hours (2 hr before chamber exposure to filtered air): Venous blood was collected, BP was recorded, and the diameter and flow-mediated dilation (FMD) of the brachial artery were measured by ultrasound. The same procedures were repeated immediately after the 2-hr filtered-air exposure (Post) and again the next morning at approximately 0800 hours (Follow-up). These latter measurements also served as the preexposure values for the CAP exposure. At approximately 1000 hours on the second day, participants were exposed to CAP for 2 hr. As with the filtered-air exposure, post and follow-up measurements were obtained immediately after CAP exposure and beginning at 0800 hours the next morning.

*Dietary supplementation*. As described previously (Tong et al. 2012), all participants were asked to restrict their intake of foods containing n-3 and n-9 FA for the 2-week period prior to and during the 4-week dietary supplementation period. Thirteen participants received 3 g/day (three 1-g capsules daily) of OO, 16 participants received 3 g/day (three 1-g capsules daily) of marine-derived n-3 FA (FO), and 13 participants were not supplemented (naive) for 28 days prior to the filtered-air exposure day. The participants were supplemented with OO or FO up to and including the days of exposure. FO and OO assignments were made using a randomized, double-blinded study design. Participants were also asked to keep 3-day food records during the second and fourth weeks of the supplementation period to assess compliance with the dietary restrictions. Nutrition Data System for Research software (NDSR; University of Minnesota) was used to analyze the food records and estimate intakes of nutrients that may confound n-3 FA. Each 1-g OO capsule contained < 1% of n-3 FA, with 73% of the FA content being oleic acid and 12% palmitic acid. Each 1-g FO capsule contained approximately 65% n-3 FA, consisting of 410 mg EPA (eicosapentaenoic acid) and 274 mg of DHA (docosahexaenoic acid). Pharmavite LLC kindly provided the OO and FO supplements. All capsules used in this study were derived from single lots. The ratios of the major plasma FAs were measured at the end of the supplementation period to determine whether ratios were consistent with expectations for the FO and OO groups, and the data has been reported previously (Tong et al. 2012).

*Controlled exposure*. CAP was generated as described previously (Tong et al. 2012) by drawing ambient air from above the roof of the HSF in Chapel Hill, North Carolina, and passing it through a two-stage aerosol concentrator capable of producing up to a 30-fold increase in particle number and mass. Air temperature and humidity were controlled inside the chamber. The concentration of particles delivered to the chamber varied with the level of naturally occurring ambient particles in the Chapel Hill airshed at the time of the exposure. Particle mass and number concentrations at the chamber inlet were monitored continuously as described previously (Samet et al. 2009). A particle dilution system was used to limit the maximal particle concentration and prevent it from exceeding 600 μg/m^3^ for > 6 min at any time during exposure. A face mask was used to assure concordance between the PM concentration in the chamber and the concentration actually inhaled by the participant. Teflon filter samples were also obtained and analyzed for particle mass gravimetrically.

The 2-hr average fine/ultrafine CAP mass concentration in the chamber was 253 ± 16 μg/m^3^ (mean ± SE). The PM_2.5_ concentration and particle number during the 2-hr CAP exposure, as well as particle size, is reported in Supplemental Material, Table S1 and Figure S1. An estimated 75% of particles were in the ultrafine range during exposure. The maximum 2-hr average inhalation exposure in this study is comparable to approximately 24-hr exposure to PM_2.5_ at the current 24-hr National Ambient Air Quality Standard (NAAQS) (U.S. EPA 2012). No clinically significant events associated with CAP exposure were observed in any of the study participants.

*Brachial artery ultrasound*. As described previously (Guthikonda et al. 2007; Schneider et al. 2008), FMD was measured by brachial artery ultrasound in the University of North Carolina Hospitals Clinical Translational Research Center (CTRC) using a 12.5-MHz imaging probe interfaced with an ATL HDI 5000 ultrasound machine (Philips), or on-site at the U.S. Environmental Protection Agency HSF using a 15-MHz imaging probe interfaced with an Acuson Sequoia 512 ultrasound machine (Siemens). The participants were transported from the HSF to the UNC-CTRC by an E85-fueled vehicle. Baseline images of the right brachial artery were captured at end diastole. The FMD was assessed after reactive hyperemia induced by inflating a pneumatic tourniquet applied distal to the antecubital fossa to a suprasystolic pressure for 5 min (Guthikonda et al. 2007). Hyperemic images were captured for 90 sec following cuff deflation. Brachial arterial diameter (BAD) was measured at baseline and at maximum dilation with customized software that utilizes edge-detection technology (Vascular Research Tools, version 5.9.0; Medical Imaging Applications).

*BP measurements*. BP was measured using a validated ambulatory BP monitor (Oscar-2; SunTech Medical) at 15-min intervals during 2 hr of exposure and at 30-min intervals before and after filtered-air and CAP exposure. A single reading at each time point was acquired and used in the analysis.

*Measurement of endothelin-1 and markers of fibrinolysis and inflammation*. Venous blood was obtained 2 hr before, immediately after, and 20 hr after each exposure. Anticoagulated plasma samples were stored at –80°C until assayed in our laboratory using commercially available ELISA kits to quantify levels of fibrinolysis markers including tissue-type plasminogen activator (tPA) and plasminogen (Enzyme Research Laboratories); D-dimer and von Willebrand factor (vWF) (Diagnostisca Stago); plasminogen activator inhibitor-1 (PAI-1) (DakoCytomation); inflammatory markers including fibrinogen (Diasorin), interleukin (IL)-6, IL-8, tumor necrosis factor alpha (TNFα), and C-reactive protein (CRP) (Meso Scale Discovery); vascular inflammatory markers including vascular cell adhesion molecule 1 (VCAM-1) and intercellular adhesion molecule 1 (ICAM-1) (Meso Scale Discovery); and vasoconstrictor endothelin-1 (Bachem Group).

*Statistical analysis*. To evaluate changes between CAP and filtered-air exposures within participants, as well as differences between the OO, FO, and naive groups, we used a two-factor (supplement and CAP concentration) mixed-effects model with a participant-specific random intercept. Changes within individuals were evaluated at two time points separately: immediately after and approximately 20 hr after exposure to CAP and filtered air, denoted “Post” and “Follow-up,” respectively. Prior to the analysis, we normalized all Post and Follow-up outcomes to their preexposure baseline (Post/Pre, Follow-up/Pre) to control for day-to-day variability. The exposure variable PM was treated as continuous, and changes between CAP and filtered-air exposures were expressed as percent point differences per 100-μg/m^3^ increase in CAP concentration relative to baseline, with the associated 95% confidence interval (CI). To test differences in baselines between the three groups and differences between exposures we used a two-factor mixed-effects model [exposure = (before filtered air, before CAPs), group = (OO, FO, naive)]. There were more females than males in each dietary supplement group in this study, and the ratio of females to males varied (9:4, 12:4, and 11:2 in OO, FO, and naive groups, respectively). However, the great majority of female participants (29 of 32) were postmenopausal, and only 4 were on hormone replacement therapy (Table 1). To examine the influence of sex on responses to CAP and the outcomes, we conducted sensitivity analyses for selected end points by adjusting for sex as a confounder and by restricting analyses to women only. We did not have sufficient power to evaluate effect modification of associations with CAP, or of differences according to supplement group, by sex. We also examined the sensitivity of results with respect to medication usage by excluding 5 participants taking statins or ACE (angiotensin-converting enzyme) inhibitors. R statistical software (version 2.15.0; R Core Team 2012) was used for analysis, and a *p-*value < 0.05 was considered significant.

**Table 1:** Characteristics of the participants before dietary restriction and supplementation.

Characteristic	Olive oil (*n* = 13)	Fish oil (*n* = 16)	Naive (*n* = 13)
Sex (male/female; *n*)	4/9	4/12	2/11
Age (years)	59.3 ± 1.1	57.4 ± 1.4	57.8 ± 1.3
Post­menopausal (*n*)	8	11	10
Race (white/black; *n*)	11/2	10/6	10/3
BMI (kg/m^2^)	26.3 ± 1.3	27.6 ± 1.1	24.9 ± 1.2
Systolic blood pressure (mmHg)	123 ± 3	122 ± 3	121 ± 2
Diastolic blood pressure (mmHg)	77 ± 2	77 ± 2	76 ± 3
Heart rate (bpm)	71 ± 3	74 ± 2	66 ± 3
Cholesterol
Total (mg/dL)	214 ± 7	201 ± 12	210 ± 10
LDL (mg/dL)	125 ± 9	117 ± 10	120 ± 9
VLDL (mg/dL)	19 ± 3	19 ± 2	18 ± 2
HDL (mg/dL)	70 ± 6	64 ± 4	71 ± 6
Triglyceride (mg/dL)	94 ± 14	97 ± 10	93 ± 8
Glucose (mg/dL)	93 ± 2	88 ± 3	92 ± 2
WBC (× 10^3^/μL)	5.46 ± 0.39	5.53 ± 0.25	5.21 ± 0.36
RBC (× 10^6^/μL)	4.76 ± 0.17	4.45 ± 0.12	4.54 ± 0.07
Platelets (× 10^3^/μL)	223.3 ± 12.2	245.9 ± 13.6	250.4 ± 13.6
Neutrophils (%)	55.3 ± 2.7	55.9 ± 2.9	50.5 ± 4.7
Lymphocytes (%)	34.6 ± 2.5	34.9 ± 2.9	29.8 ± 3.2
Smoking history (*n*)
Nonsmokers	11	11	13
Ex-smokers	2	5	0
Current smokers	0	0	0
Medications (*n*)
Statin	2	1	0
β-Adrenergic receptor blockers	0	0	0
ACE inhibitors	3	2	0
Antidepressant	1	2	0
NSAIDs	0	0	0
HRT	1	2	1
Data are mean ± SEM. Age, BMI, medication usage, blood pressure, blood cell counts, serum glucose, and lipids were not significantly different among the groups (ANOVA, *p* > 0.05). Abbreviations: ACE inhibitors, angiotensin-converting enzyme inhibitors; HDL, high-density lipoprotein; HRT, hormone replacement therapy; LDL, low-density lipoprotein; NSAIDs, nonsteroidal anti-inflammatory drugs; RBC, red blood cells; VLDL, very low density lipoprotein; WBC, white blood cells.			

## Results

Table 1 presents characteristics of the research participants. Age, body mass index (BMI), smoking history, medication usage, BP, serum glucose, and lipids did not differ statistically among the groups. Before starting supplementation with OO or FO, or no supplements (naive), all participants reported low dietary intakes of foods rich in n-3 and n-9 FA (Tong et al. 2012). Participants showed good compliance with their adherence to the dietary restriction and OO or FO supplementation schedule (Tong et al. 2012).

*Endothelial function*. The average baseline diameter and FMD of brachial artery measured before, immediately after, and 20 hr after CAP exposure are presented in Supplemental Material, Table S2. Compared with the naive group, the mean value of FMD measured prior to the filtered-air exposure was higher in the FO group (7.19 ± 0.88 vs. 6.57 ± 0.65; *p* = 0.08) when analyzed using a two-factor mixed-effects linear model for differences in baselines between exposures (filtered air, CAP) and supplemental groups (OO, FO, naive). There were no significant differences among the pre-filtered air–exposure diameter mean values in the OO, FO, and naive groups.

**Figure 1:**
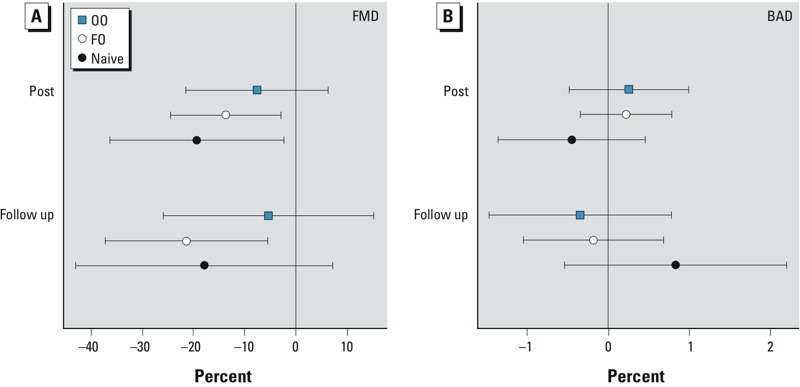
Effect of concentrated ambient particle (CAP) exposure on parameters of brachial artery ultrasound. Flow-mediated dilation (FMD; *A*) and baseline diameter (BAD; *B*) of the brachial artery were measured by ultrasound before, immediately after exposure to filtered-air and CAP (post), and again the next morning (follow-up) as described in “Methods.” Symbols represent differences between CAP and filtered-air exposure, expressed as average percent point differences per 100-μg/m^3^ increase in CAP, relative to the baseline; bars indicate 95% CIs.

The mean percent difference in FMD and baseline BAD per 100-μg/m^3^ increase in CAP concentration immediately after and 20 hr after CAP exposure relative to filtered-air control is shown in Table 2. FMD was significantly lower immediately after CAP exposure in the naive group (–19.4% average decrease relative to baseline per 100-μg/m^3^ increase in CAP concentration; 95% CI: –36.4%, –2.3%; *p* = 0.03) and also in the FO group (–13.7%; 95% CI: –24.5%, –2.9%; *p* = 0.01) (Table 2 and Figure 1). In the OO group, however, exposure to CAP resulted in a smaller nonsignificant decrease in FMD (–7.6%; 95% CI: –21.5%, 6.3%; *p* = 0.27). In the FO group, FMD 20 hr after CAP exposure was still significantly lower than baseline (–21.5%; 95% CI: –37.3%, –5.6%; *p* = 0.01). Differences between the groups were not significant. Reduction in FMD was persistent after adjusting for confounding by sex as well as in the “female-only” analysis (see Supplemental Material, Table S3). There was also no significant difference in BAD either immediately after or 20 hr after CAP exposure in any of the groups (Figure 1 and Table 2).

**Table 2:** Mean percent point difference per 100-μg/m^3^ increase in CAP concentration relative to baseline (before filtered-air exposure) measured immediately after (post) and 20 hr after (FU) CAP exposure in each group.

End point	OO (*n* = 13)		FO (*n* = 16)		Naive (*n* = 13)	
	Post-CAP	Follow-up	Post-CAP	Follow-up	Post-CAP	Follow-up
BAU
FMD	–7.6 (–21.5, 6.3)	–5.4 (–25.8, 15.1)	–13.7 (–24.5, –2.9)*	–21.5 (–37.3, –5.6)*	–19.4 (–36.4, –2.3)*	–17.9 (–43.0, 7.2)
BAD	0.2 (–0.5, 0.9)	–0.3 (–1.4, 0.8)	0.2 (–0.3, 0.8)	–0.2 (–1.0, 0.7)	–0.4 (–1.3, 0.4)	0.8 (–0.5, 2.2)
Blood markers
ET-1	–4.7 (–14.8, 5.4)	–10.0 (–22.1, 2.1)	–0.4 (–7.8, 7.0)	1.4 (–7.9, 10.8)	8.3 (–3.7, 20.2)	17.1 (2.2, 32.0)*
tPA	11.6 (0.8, 22.2)*	10.9 (0.1, 21.8)*	2.2 (–6.4, 10.9)	1.9 (–7.2, 11.1)	–0.50 (–12.8, 11.7)	1.2 (–11.7, 14.0)
PAI-1	0.1 (–6.6, 6.8)	–4.4 (–15.5, 6.6)	–0.6 (–5.6, 4.4)	–2.3 (–10.9, 6.3)	6.3 (–1.7, 14.4)	6.2 (–7.4, 19.7)
D-dimer	–4.1 (–24.0, 15.8)	–11.6 (–22.6, –0.5)*	–1.9 (–16.5, 12.6)	1.4 (–7.2, 10.0)	–16.9 (–40.4, 6.7)	–2.9 (–16.5, 10.6)
Plasminogen	–6.7 (–16.3, 2.9)	–5.3 (–14.3, 3.6)	–3.1 (–10.4, 4.2)	1.2 (–5.7, 8.2)	–2.0 (–13.6, 9.6)	–3.7 (–14.6, 7.3)
vWF	4.5 (–2.5, 11.7)	0.4 (–20.7, 21.5)	0.3 (–4.9, 5.5)	7.0 (–9.4, 23.4)	–0.1 (–8.5, 8.3)	–0.6 (–26.5, 25.2)
Fibrinogen	1.0 (–3.8, 5.8)	0.4 (–4.0, 4.8)	0.2 (–3.4, 3.8)	2.7 (–0.7, 6.2)	5.0 (–0.8, 10.8)	2.3 (–3.1, 7.8)
CRP	0.7 (–24.4, 25.9)	3.7 (–14.4, 21.8)	–1.7 (–20.7, 17.2)	–5.0 (–19.1, 9.1)	–2.5 (–32.8, 27.7)	9.5 (–12.7, 31.7)
ICAM-1	0.8 (–29.1, 30.7)	–0.5 (–17.4, 16.3)	–1.8 (–24.3, 20.6)	–1.7 (–14.8, 11.3)	0.3 (–35.6, 36.3)	7.3 (–13.3, 27.9)
VCAM-1	0.1 (–28.4, 28.7)	–0.7 (–20.0, 18.6)	–2.4 (–23.9, 19.0)	–3.2 (–18.2, 11.8)	2.1 (–32.1, 36.5)	5.5 (–18.1, 29.2)
IL-6	–1.4 (–12.3, 9.3)	–1.0 (–12.3, 10.2)	–3.9 (–12.2, 4.3)	–1.7 (–10.5, 7.0)	–4.7 (–17.7, 8.4)	–9.2 (–23.0, 4.6)
IL-8	–1.1 (–5.7, 3.4)	1.2 (–4.0, 6.5)	–0.1 (–3.6, 3.4)	0.3 (–3.8, 4.4)	3.7 (–1.8, 9.2)	–3.3 (–9.8, 3.1)
TNFα	1.8 (–1.5, 5.2)	0.2 (–3.5, 4.0)	–0.04 (–2.6, 2.5)	0.6 (–2.3, 3.5)	–0.3 (–4.3, 3.7)	–3.9 (–8.6, 0.6)
End points are summarized as mean and 95% confidence interval. Abbreviations: BAD, baseline diameter of brachial artery; BAU, brachial artery ultrasound; CAP, concentrated ambient air pollution particles; CRP, c-reactive protein; ET-1, endothelin-1; FMD, flow-mediated dilation; FO, fish oil group; ICAM-1, intercellular adhesion molecule 1; Il, interleukin; OO, olive oil group; PAI-1, plasminogen activator inhibitor-1; TNFα, tumor necrosis factor α; tPA, tissue-type plasminogen activator; VCAM-1, vascular cell adhesion protein 1; vWF, von Willebrand factor. *p < 0.05, compared with pre-CAP.						

*Blood pressure*. There was no significant difference in BP before filtered-air exposure between the OO, FO, and naive groups (Table 1). There were statistically nonsignificant increases in systolic BP 30 min after CAP exposure in the naive group (+2.5 mmHg per 100-μg/m^3^ increase in CAP concentration compared with filtered-air exposure; *p* = 0.09) and 60 min after exposure in the FO (+2.7 mmHg; *p* = 0.06) and OO (+2.1 mmHg; *p* = 0.22) groups. Diastolic BP was significantly increased 30 min after exposure in the naive group (+2.1 mmHg per 100-μg/m^3^ increase in CAP concentration compared with filtered-air exposure; *p* = 0.04) and 60 min after exposure in the FO (+2.1 mmHg; *p* = 0.008) and OO (+2.1 mmHg per 100-μg/m^3^ increase in CAP concentration compared with filtered-air exposure; *p* = 0.03) groups.

*Blood markers of fibrinolysis*. The average concentrations of blood markers of fibrinolysis at baseline, immediately postexposure, and 20 hr postexposure are presented in Supplemental Material, Table S4. In all three supplement groups, we observed higher levels of plasma PAI-1 in the morning (0800–0900 hours) and lower levels in the afternoon (1300–1400 hours). The observed variation is consistent with previous reports (Paschos and FitzGerald 2010; Rudnicka et al. 2007) and may indicate diurnal variation in levels of plasma PAI-1; however, this study was not designed to further examine this hypothesis. the baseline tPA concentration (i.e., before filtered-air exposure) was significantly lower in the OO group compared with the naive group, and baseline PAI-1 and plasminogen concentrations were significantly lower in the OO and FO groups compared with the naive group (see Supplemental Material, Table S4), possibly due to effects of OO and FO supplementation (Mehta et al. 1988; Pérez-Jiménez et al. 2006).

**Figure 2:**
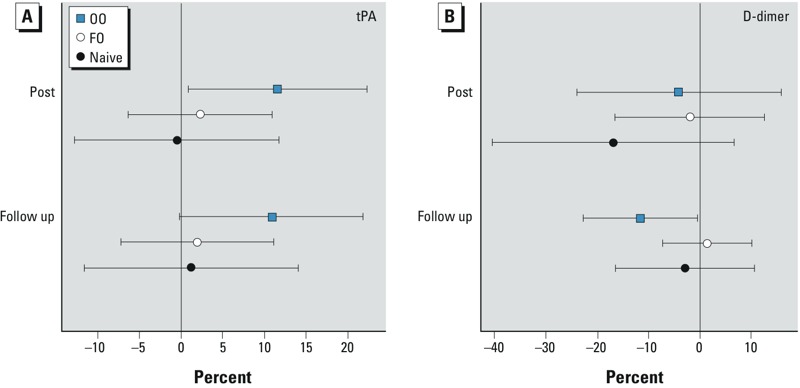
Effect of concentrated ambient particle (CAP) exposure on plasma markers of fibrinolysis. Blood was collected before and immediately after exposure (post) to filtered air and CAP, and again the next morning (follow-up), and markers of fibrinolysis, tPA (*A*) and D-dimer (*B*), were assayed by ELISA as described in “Methods.” Symbols represent differences between CAP and filtered-air exposure, expressed as average percent point differences per 100-μg/m3 increase in CAP, relative to the baseline; bars indicate 95% CIs.

In the OO group, the average plasma concentration of tPA increased immediately after CAP exposure in the OO group (11.6%; 95% CI: 0.8%, 22.2%; *p* = 0.04), and this increase persisted for 20 hr, at which time there was a 10.9% increase (95% CI: 0.1%, 21.8%; *p* = 0.05) for this group (Table 2 and Figure 2). In addition, plasma D-dimer levels were 11.6% lower (95% CI: –22.6%, –0.5%; *p* = 0.04) 20 hr after CAP exposure in the OO group.

The association between CAP exposure and tPA in the OO group was robust to adjustment for confounding by sex, but changed from positive to slightly negative when the four men in the OO group were excluded from the analysis, suggesting substantial differences in association between CAP and tPA among males and females in the OO group (see Supplemental Material, Table S3). The association between CAP and D-dimer levels in the OO group was similar after adjusting for sex and after excluding men from the analysis (see Supplemental Material, Table S3). When five participants taking either statins or ACE inhibitors were excluded from the OO group, plasma D-dimer levels were no longer significantly decreased 20 hr after CAP exposure (–0.7% per 100-μg/m^3^ increase in CAP; 95% CI: –10.4%, 9%; *p* = 0.88). This suggests that these medications may have influenced effects of CAP on plasma D-dimer levels, although differences due to chance cannot be ruled out because of small sample size and potential for selection bias. Excluding participants using these medications did not have obvious impact on other associations in the OO group, nor did it appear to influence associations in FO or naive groups.

**Figure 3:**
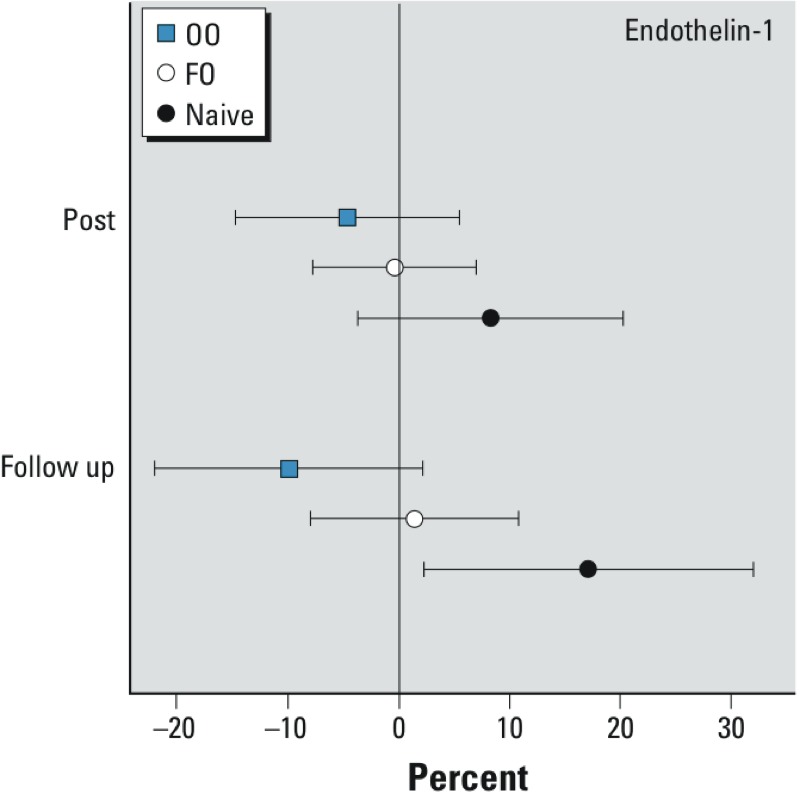
Effect of concentrated ambient particle (CAP) exposure on plasma endothelin-1 levels. Blood was collected before and immediately after exposure (post) to filtered air and CAP, and again the next morning (follow-up) and assayed for endothelin-1 by ELISA as described in “Methods.” Symbols represent differences between CAP and filtered-air exposure, expressed as average percent point differences per 100-μg/m3 increase in CAP, relative to the baseline; bars indicate 95% CIs.

*Endothelin-1 level*. Blood concentrations of endothelin-1, a potent vasoconstrictor, could mediate the vascular effects of CAP exposure. There was an increase in plasma endothelin-1 levels in the naive group (17.1%; 95% CI: 2.2%, 32.0%; *p* = 0.02) 20 hr after CAP exposure, but not in the OO group (Figure 3 and Table 2).

*Markers of inflammation*. Before exposure to filtered air, levels of IL-8, ICAM-1, and VCAM-1 were significantly lower in the OO and FO groups relative to the naive group, whereas TNFα levels were higher in the OO and FO groups relative to the naive group (see Supplemental Material, Table S4). There were no significant changes in concentrations of plasma markers of inflammation after CAP exposure in the OO, FO, and naive groups (Table 2).

## Discussion

In the present study, acute exposure to CAP was associated with decreased endothelial function, as measured by FMD, in middle-aged volunteers who did not receive a dietary supplement. We further show that dietary supplementation with OO, but not FO, appeared to blunt the endothelial dysfunction induced by exposure to CAP. We also observed that the concentrations of the blood fibrinolysis marker tPA increased immediately after CAP exposure in participants supplemented with OO, an effect that persisted for 20 hr after exposure. In addition, plasma endothelin-1 levels were increased 20 hr after CAP exposure in the unsupplemented group but not in the OO group. Taken together, these findings suggest that supplementation with OO provides potentially beneficial vascular effects by conferring protection against CAP-induced endothelial dysfunction in middle-aged adults.

Endothelial dysfunction is an important factor in the development of hypertension and atherosclerosis that is associated with an increased risk of coronary heart disease. Specifically, endothelial dysfunction induced by air pollution exposure has been implicated as an important mechanism in the development of cardiovascular diseases (Brook et al. 2010; Krishnan et al. 2013). Mechanistically, oxidative stress and inflammation are two mechanisms that have been associated with the adverse health effects of air pollution exposure. It has been suggested that antioxidant components in certain food groups can limit oxidative damage and thereby protect endothelial function (Moreno and Mitjavila 2003). The results presented here suggest that dietary supplementation with OO may prevent deleterious effects of CAP exposure on vascular function and might, therefore, represent a practical approach to reduce the mortality and morbidity of cardiovascular diseases associated with PM exposure.

There was a smaller CAP-induced reduction in FMD in participants supplemented with OO compared with FO-supplemented and unsupplemented participants in this study, supporting a role for OO in protecting vascular function from PM-induced health effects. The oleic acid in OO has antioxidant and anti-inflammatory properties (Perona et al. 2006), and OO supplementation has been shown to improve endothelial function and lipid profile (Zern and Fernandez 2005) and lower the risk of coronary heart disease events (Estruch et al. 2013; Hertog et al. 1995). Two months of dietary supplementation with OO was previously associated with improved endothelial function and a decrease in BP in a study of young women with mild hypertension (Moreno-Luna et al. 2012). Furthermore, in a clinical trial in healthy young adults, 16 weeks of supplementation with 4 g/day of OO led to an increase in FMD (Singhal et al. 2013). Our finding suggests that 4 weeks of dietary OO supplementation attenuate PM-induced reductions in FMD, possibly due to the effect of OO to protect against the adverse effects of PM exposure on endothelial function.

We also observed in this study that, on average, participants in the OO supplementation group had significant changes in the levels of tPA to CAP exposure, whereas those in FA and naive groups did not. There was a significant increase in plasma levels of tPA immediately after CAP exposure, and this effect persisted 20 hr postexposure in the OO group. tPA is a serine protease that catalyzes the conversion of plasminogen to plasmin, the major enzyme responsible for the breakdown of thrombi. In contrast to tPA, D-dimer was significantly reduced 20 hr after CAP exposure in the OO group, although the effect was no longer present after eight participants using statins and ACE inhibitors were excluded from the OO group, which suggests that the association may have been influenced by medication use in these participants. D-dimer is a fibrin degradation product present in the blood after a thrombus is degraded by fibrinolysis. We have previously reported that the levels of D-dimer were increased following controlled exposure of young healthy volunteers to ultrafine CAP (Samet et al. 2009). The increase in tPA level suggests that the supplementation with OO may activate the fibrinolysis system that protects against thrombosis induced by CAP exposure. However, it is difficult to reconcile this mechanism with the simultaneous decrease in D-dimer level observed in these participants. One possibility is that OO could be decreasing prothrombotic factors that were not measured in this study.

In addition, we observed that plasma levels of endothelin-1 were increased after CAP exposure in the unsupplemented group but not in participants supplemented with OO or FO. Several studies have shown increased plasma endothelin-1 levels after controlled exposure of human volunteers to diesel exhaust (Peretz et al. 2008) and following exposure to urban ambient levels of air pollution in adults (Liu et al. 2009) and children (Calderón-Garcidueñas et al. 2007), suggesting that increased concentrations of this potent vasoconstrictor is a consequence of endothelial damage resulting from air pollution exposure. The attenuation of CAP-induced endothelin-1 levels in the OO group shown in this study suggests beneficial effects of OO supplementation on vascular tone and endothelial function. However, increases in BP following CAP exposure were comparable among the OO, FO, and unsupplemented groups. It has been suggested that the CAP-induced BP elevation may be caused by autonomic imbalance (Brook et al. 2009). We previously demonstrated that CAP exposure alters the sympathovagal balance (Samet et al. 2009; Tong et al. 2012) by increasing the sympathetic input to the cardiovascular system, which could result in increased BP.

It has been hypothesized that FO-mediated enhancement of endothelial function is a potential strategy by which the population can be protected from the adverse effects of PM. Previous studies have demonstrated that FO (EPA and DHA) improve systemic arterial compliance in individuals with dyslipidemia (Nestel et al. 2002) and improve endothelial function in individuals with dyslipidemia, heart failure, and diabetes (Duda et al. 2009b; Saravanan et al. 2010). However, trials of FO supplementation in healthy volunteers have shown inconsistent results (Khan et al. 2003; Singhal et al. 2013). One study found that 8 months of FO supplements improved endothelial function in middle-aged men and women (Khan et al. 2003). By contrast, 16 weeks of DHA supplementation had no effect on indices of endothelial function in a study of healthy young volunteers (Singhal et al. 2013). In general, studies have shown that EPA and DHA levels in blood are not linked to FMD in normal healthy adults (Egert and Stehle 2011; Leeson et al. 2002; Sanders et al. 2011; Saravanan et al. 2010; Singhal et al. 2013). Consistent with these reports, in the present study we did not observe a significant benefit of 4 weeks of FO supplementation on CAP-induced reduction in FMD.

The mechanism by which OO supplementation attenuates CAP-induced vascular effects was not investigated in this study. It is possible that OO supplementation increases the bioavailability of plasma nitrites/nitrates, which in turn improves endothelial function (Moreno-Luna et al. 2012). In the present study, OO supplementation appeared to blunt the CAP-induced reduction in FMD, consistent with an effect of OO on the bioavailability of NO. Another function of OO is to promote anti-inflammatory effects in the vasculature. It has been shown that oleic acid, a principal bioactive component of OO, inhibits endothelial activation by suppressing inflammatory responses *in vitro* (Carluccio et al. 1999). It is also possible that OO supplementation increases resistance of HDL (high-density lipoprotein) and LDL (low-density lipoprotein) to oxidation (Moreno et al. 2003; Solà et al. 1997) induced by CAP exposure.

In order to avoid potential variations in FA equilibrium times and particle washout periods, this study did not use a standard crossover design involving a randomized exposure to air and CAP. In addition, conclusions derived from the small number of participants included in the study may not be applicable to the population as a whole. Furthermore, the modest sample size and the number of secondary end points measured, which could inflate the significance of the findings, are additional statistical limitations. In spite of these limitations, as the first controlled exposure study of this type, the present study reports statistically significant differences between the response to CAPs and filtered air under each of the three dietary regimens for several important outcomes. These findings provide the basis for a future cohort study focusing on between-group comparisons in order to confirm and further evaluate OO supplementation as a protective intervention against the adverse health effects of PM inhalation.

## Conclusion

In this study we observed that a 2-hr exposure to CAP impaired vascular endothelial function for up to 20 hr, and that dietary supplementation with OO, but not FO, attenuated the FMD reduction induced by CAP exposure. Further, supplementation with OO seemed to alter blood markers associated with fibrinolysis and vasoconstriction in PM-exposed human volunteers. These data, therefore, suggest that OO supplementation should be further examined as a possible intervention to protect against the adverse vascular effects of air pollution exposure.

## Supplemental Material

(271 KB) PDF
