# Supplementary material for: Dietary Supplementation with Olive Oil or Fish Oil and Vascular Effects of Concentrated Ambient Particulate Matter Exposure in Human Volunteers
Source: Environ Health Perspect. 2015 May 1;123(11):1173–9. doi: 10.1289/ehp.1408988 (PMC4629741; doi:10.1289/ehp.1408988)
Supplement: (271 KB) PDF [file ehp.1408988.s001.acco.pdf]

Environ Health Perspect
doi: 10.1289/ehp.1408988

Note to Readers: *EHP* strives to ensure that all journal content is accessible to all readers. However, some figures and Supplemental Material published in *EHP* articles may not conform to 508 standards due to the complexity of the information being presented. If you need assistance accessing journal content, please contact ehp508@niehs.nih.gov. Our staff will work with you to assess and meet your accessibility needs within 3 working days.

Supplemental Material

Dietary Supplementation with Olive Oil or Fish Oil and Vascular Effects of Concentrated Ambient Particulate Matter Exposure in Human Volunteers

Haiyan Tong, Ana G. Rappold, Melissa Caughey, Alan L. Hinderliter, Maryann Bassett, Tracey Montilla, Martin W. Case, Jon Berntsen, Philip A. Bromberg, Wayne E. Cascio, David Diaz-Sanchez, Robert B. Devlin, and James M. Samet

Table of Contents

Table S1. $PM_{2.5}$ mass, particle numbers and particle size of CAP exposure.

Table S2. Average values of brachial artery ultrasound endpoints.

Table S3. Sensitivity analysis of selected endpoints for confounding by gender.

Table S4. Average concentrations of blood markers.

Figure S1. Particle size distribution curve during 2 hrs exposure. Particle size was measured by TSI scanning mobility particle sizer (SMPS) continuously during exposure. Particle size was averaged from the 42 exposures in this study.

Table S1. $PM_{2.5}$ mass, particle numbers and particle size of CAP exposure.

Values	$PM_{2.5}$ mass (μg/m^3)	particle number	particle size (NMD, μm)*	particle size (MMD, μm)#
Minimum	67.9	22,634	0.033	0.33
$IQR_{25\%}$	177.5	66,992	0.041	0.345
Median	243.2	152,508	0.051	0.37
$IQR_{75\%}$	322.2	214,432	0.070	0.415
Maximum	470.3	1,971,000	0.098	0.56

NMD, number based median particle diameter; MMD, mass based median particle diameter. *Particle size (NMD; 50% of the total number of particles are smaller than the given size) was determined on particles count during 2-hr exposure; 75% particles during exposure were <0.1 μm. #Particle size (MMD) measured during external chamber audits and not during exposures; 96% of particles were <2.5 μm.

Table S2. Average values of brachial artery ultrasound endpoints.

End-Points	Olive Oil (n=13)					Fish Oil (n=16)					Naive (n=13)				
	Filtered Air		CAP			Filtered Air		CAP			Filtered Air		CAP		
	Before	After	Before	After	FU	Before	After	Before	After	FU	Before	After	Before	After	FU
BAD, mm	3.18±0.15	3.12±0.15	3.18±0.16	3.13±0.16	3.14±0.16	3.24±0.12	3.24±0.13	3.22±0.12	3.22±0.13	3.16±0.14	3.06±0.15	3.12±0.15	3.18±0.16	3.13±0.16	3.14±0.16
FMD,%	6.57±0.65	8.27±0.84	7.77±0.69	8.58±0.99	8.50±0.82	7.19±0.88	7.84±0.88	8.51±0.86	7.63±1.02	7.85±1.11	5.28±0.67	5.97±0.78	5.86±0.56	4.46±0.56	4.85±0.59

Values are mean ± SEM. BAD, brachial artery diameter; FMD, flow-mediated dilatation.

Table S3. Sensitivity analysis of selected endpoints for confounding by gender.

Endpoints		Non-adjusted		Adjusted		Female only	
		**Mean (95% CI)**	**p**	**Mean (95% CI)**	**p**	**Mean (95% CI)**	**p**
FMD (post-CAP)	OO	-7.6(-21.5, 6.3)	0.273	-7.5(-21.3, 6.3)	0.281	-4.5(-23.5, 14.6)	0.635
	FO	-13.7(-24.5, -2.9)	0.014	-13.4(-24.1, -2.7)	0.015	-16.3(-30.5, -2.1)	0.026
	naive	-19.4(-36.4, -2.3)	0.026	-19.4(-36.3, -2.5)	0.026	-20.1(-40.3, 0.1)	0.051
ET-1 (FU-CAP)	OO	-10.0(-22.1, 2.1)	0.103	-9.9(-22.0, 2.2)	0.106	-8.2(-25.2, 8.7)	0.329
	FO	1.4(-7.9, 10.8)	0.762	1.6(-7.8, 10.9)	0.732	0.21(-12.5, 12.9)	0.972
	naive	17.1(2.2, 32.0)	0.025	17.1(2.2, 31.9)	0.025	15.5(-2.6, 33.5)	0.090
t-PA (post-CAP)	OO	11.6(0.8, 22.2)	0.035	11.5(0.7, 22.2)	0.036	-3.2(-13.8, 7.2)	0.528
	FO	2.2(-6.4, 10.9)	0.595	2.4(-6.2, 11.0)	0.580	3.8(-3.5, 11.1)	0.292
	naive	-0.50(-12.8, 11.7)	0.934	-0.5(-12.8, 11.7)	0.929	-4.5(-14.4, 5.3)	0.349
D-dimer (FU-CAP)	OO	-11.6(-22.6, -0.5)	0.040	-11.6(-22.7, -0.4)	0.042	-13.0(-27.4, 1.4)	0.075
	FO	1.4(-7.2, 10.0)	0.741	1.4(-7.2, 10.0)	0.739	3.1(-7.6, 13.9)	0.554
	naive	-2.9(-16.5, 10.6)	0.665	-2.9(-16.6, 10.7)	0.667	-3.4(-18.7, 11.8)	0.649

Endpoints are summarized as mean percent point difference per 100 μg/m^3 of CAP exposure relative to baseline (pre-filtered-air exposure) measurements and 95% confidence intervals. OO, olive oil group; FO, fish oil group; CAP, concentrated ambient air pollution particles; FU, follow-up; FMD, flow-mediated dilatation; ET-1, endothelin-1; tPA, tissue-type plasminogen activator.

Table S4. Average concentrations of blood markers.

End-Points	Olive Oil (n=13)					Fish Oil (n=16)					Naive (n=13)				
	Filtered Air		CAP			Filtered Air		CAP			Filtered Air		CAP		
	Before	After	Before	After	FU	Before	After	Before	After	FU	Before	After	Before	After	FU
ET-1, pg/mL	81.9±11.6	80.6±11.5	85.3±10.6	69.3±10.5	76.5±9.6	110.5±23.2	110.3±23.9	99.2±18.1	104.8±25.8	99.6±27.0	83.9±17.0	78.5±15.7	88.9±22.5	102.0±27.6	118.7±29.8
tPA, ng/mL`	1.79±0.33*	1.93±0.29	1.67±0.34	1.95±0.36	1.56±0.20	6.26±2.53	6.23±2.37	5.03±1.78	5.26±1.77	3.84±1.53	7.55±1.02	7.78±1.20	7.31±1.02	7.06±082	7.80±1.15
PAI-1, ng/mL	1.58±0.31*	0.89±0.18	1.55±0.35	0.77±0.15	1.43±0.38	2.53±0.36*	1.20±0.22	2.50±0.48	1.01±0.12	2.12±0.42	5.63±1.61	1.93±0.43	4.24±1.33	2.16±0.63	3.76±0.96
D-dimer, ng/mL	429±98	428±98	489±107	443±93	448±107	394±52†	383±56	424±58	372±53	415±55	262±44	379±117	243±35	323±70	234±37
Plasm, ng/mL	109.6±6.0*	131.8±8.7	125.0±9.0	120.5±6.8	121.5±5.1	120.7±5.6*	135.7±14.5	115.0±4.3	119.6±5.5	114.5±4.1	150.5±13.8	172.6±12.4	179.4±19.8	165.9±8.6	169.1±13.2
vWF, %	98.3±9.1	88.6±12.7	102.0±11.6	103.9±11.0	98.8±10.3	94.3±9.2	99.9±8.4	97.5±10.9	98.5±8.6	96.1±8.7	91.0±7.9	89.2±8.0	92.6±7.4	91.0±6.8	95.2±9.6
Fibrinogen, mg/dl	292.0±20.9	295.6±24.0	289.1±22.2	298.4±18.7	294.0±26.9	258.2±10.6	281.3±15.4	243.9±10.5	265.0±14.3	255.7±14.0	284.0±13.3	283.2±20.4	262.4±9.8	295.3±20.7	263.4±10.0
CRP, ng/ml	4106±954	4368±1046	4155±1283	4183±1326	4752±2180	1407±343	1315±291	1381±305	1295±302	1156±274	3161±960	3289±1352	2663±946	2165±612	1878±527
ICAM-1, ng/ml	307±28*	305±24	284±21	293±21	278±30	277±24*	289±31	297±32	281±29	290±33	471±101	607±147	453±105	505±159	405±74
VCAM-1, ng/ml	460±44*	459±40	438±33	434±31	433±50	391±33*	405±44	420±35	394±38	429±46	780±159	966±229	742±164	751±185	656±114
Il-6, pg/ml	1.50±0.22	1.48±0.18	1.52±0.24	1.63±0.24	1.48±0.23	1.27±0.14	1.32±0.17	1.28±0.14	1.14±0.14	1.20±0.13	1.07±0.26	1.15±0.37	1.10±0.35	0.97±0.23	1.05±0.25
IL-8, pg/ml	3.23±0.29*	2.69±0.31	3.07±0.28	2.63±0.19	3.11±0.26	3.64±0.30*	3.09±0.22	3.59±0.33	3.03±0.23	3.56±0.30	5.44±0.45	4.21±0.34	5.48±0.52	4.52±0.45	5.43±0.47
TNFα, pg/ml	5.31±0.60*	4.57±0.23	5.32±0.64	5.26±0.57	5.28±0.61	6.43±0.50*	6.16±0.49	6.28±0.50	6.05±0.53	6.43±0.51	2.77±0.16	2.73±0.15	2.82±0.20	2.79±0.21	2.86±0.23
Cholesterol	205±7	205±8	202±8	208±8	195±7	196±12	204±12	192±11	199±11	186±10	205±8	218±9	208±8	217±8	207±8
LDL, mg/dl	114±7	112±8	113±7	113±7	106±6	113±10	120±10	110±9	114±9	108±9	116±7	120±7	118±7	121±6	118±6
VLDL, mg/dl	26±2	28±3	24±2	31±4	28±4	24±3	24±3	26±3	26±3	24±3	20±2	27±4	22±2	26±3	21±2
HDL, mg/dl	65±5	65±5	64±5	64±5	61±5	59±4	61±4	57±4	59±4	54±4	69±7	71±7	68±7	70±7	67±7
TG, mg/dl	131±11	141±15	123±11	153±18	142±19	121±17	118±15	128±15	129±15	120±13	101±10	133±21	110±11	129±13	106±10

Values are mean ± SEM. ET-1, endothelin-1; tPA, tissue-type plasminogen activator; PAI-1, plasminogen activator inhibator-1; plasm, plasminogen; vWF, von Willebrand factor; CRP, c-reactive protein; ICAM-1, intercellular adhesion molecule 1; VCAM-1, vascular cell adhesion protein 1; Il-6, interleukin 6; Il-8, interleukin 8; TNFα, tumor necrosis factor α. LDL, low-density lipoprotein; VLDL, very low-density lipoprotein; HDL, high- density lipoprotein; TG, triglyceride. *$p<0.05$, †$p<0.10$, compared to pre-filtered air in the naive group.

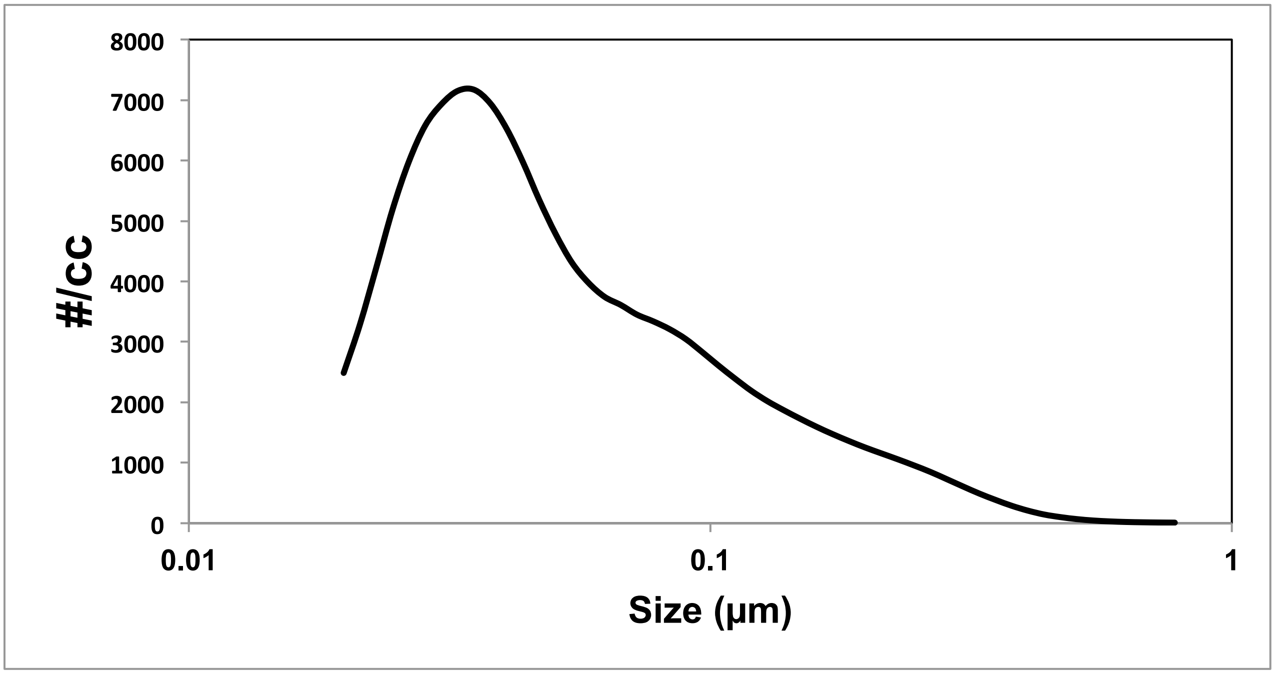

Figure S1. Particle size distribution curve during 2 hrs exposure. Particle size was measured by TSI scanning mobility particle sizer (SMPS) continuously during exposure. Particle size was averaged from the 42 exposures in this study.
